# Serum anti-POTEE antibodies as a novel serological biomarker for early detection and prognostic stratification in gastrointestinal cancers

**DOI:** 10.1186/s12885-026-16732-6

**Published:** 2026-08-10

**Authors:** Makoto Sumazaki, Satoshi Yajima, Masaaki Ito, Takashi Suzuki, Yoko Oshima, Fumiaki Shiratori, Hirotaka Takizawa, Shu-Yang Li, Bo-Shi Zhang, Yoichi Yoshida, Tomoo Matsutani, Takaki Hiwasa, Hideaki Shimada

**Affiliations:** 1 Department of Clinical Oncology, Toho University Graduate School of Medicine, Tokyo, 143-8541 Japan; 2 Department of Gastroenterological Surgery, Toho University School of Medicine, 6-11-1 Omori-Nishi, Ota-ku, 143-8541 Tokyo, Japan; 3 Port Square Kashiwado Clinic, Kashiwado Memorial Foundation, Chiba, 260-0025 Japan; 4 Department of Neurological Surgery, Graduate School of Medicine, Chiba University, Chiba, 260-8670 Japan

**Keywords:** Antibody biomarker, Esophageal squamous cell carcinoma, Gastric adenocarcinoma, Colorectal adenocarcinoma, Overall survival, POTEE, Cancer Testis antigen

## Abstract

**Background:**

Given the highly aggressive nature of gastrointestinal cancers, the identification of novel biomarkers for diagnosis and prognosis is of considerable clinical importance.

**Methods:**

Candidate cancer-testis (CT) antigens were first screened using RNA-sequencing data from normal human tissues; candidates showing high commonality across tumor RNA-sequencing datasets publicly available in The Cancer Genome Atlas were subsequently prioritized, and POTEE was identified as a promising target. Recombinant POTEE protein was expressed in Escherichia coli, purified by affinity chromatography, and used as the antigen for serological analysis.

**Results:**

Serum levels of anti-POTEE antibodies (s-POTEE-Abs) were significantly elevated in 121 patients with esophageal squamous cell carcinoma, 62 patients with gastric adenocarcinoma, and 158 patients with colorectal adenocarcinoma compared with those in 96 healthy donors. Receiver operating characteristic curve analysis showed that the diagnostic performance of s-POTEE-Ab was comparable to that of s-p53-Ab, with area under the curve values of 0.832 versus 0.829 in esophageal squamous cell carcinoma, 0.680 versus 0.698 in gastric adenocarcinoma, and 0.735 versus 0.706 in colorectal adenocarcinoma, respectively. In survival analyses, s-POTEE-Ab positivity was associated with poorer overall survival in ESCC and CRAC, but not in GAC. After multivariable adjustment, an independent prognostic association was retained only in CRAC.

**Conclusions:**

These findings suggest that s-POTEE-Ab may represent a promising exploratory serological biomarker for gastrointestinal cancers. Its prognostic relevance appears to be cancer-type dependent and requires further validation in independent cohorts.

**Supplementary Information:**

The online version contains supplementary material available at https://doi.org/10.1186/s12885-026-16732-6.

## Background

Gastrointestinal cancers rank among the most common malignancies worldwide and impose a substantial global health burden because of their high incidence and mortality [1, 2]. Although recent advances in molecular pathology have facilitated the identification of novel biomarkers and the development of targeted therapies, the prognosis of patients with gastrointestinal cancers remains poor, particularly in advanced-stage disease. The discovery of clinically useful biomarkers for cancer detection, risk stratification, and prognostic assessment therefore remains an important objective in gastrointestinal oncology [3, 4].

Cancer-testis antigens (CTAs) comprise a unique group of tumor-associated antigens characterized by their restricted expression pattern in normal tissues and aberrant expression in a wide variety of cancers. Under physiological conditions, CTAs are expressed predominantly in immune-privileged tissues such as the testis and, in some cases, the placenta, while their expression is largely absent in normal adult somatic tissues. Because germ cells in the testis do not normally provoke systemic immune responses, ectopic expression of CTAs in cancer cells can render them immunogenic. This distinctive biological feature has made CTAs attractive candidates not only as tumor- associated biomarkers but also as targets for cancer immunotherapy [5, 6]. Indeed, several CTAs, exemplified by NY-ESO-1, have been extensively investigated as diagnostically and therapeutically relevant molecules in human cancers [7, 8]. These observations suggest that CTA-related humoral immune responses may provide a valuable basis for the identification of novel serological biomarkers.

For several decades, serological identification of antigens by recombinant cDNA expression cloning (SEREX) has been employed as an effective strategy for discovering tumor-associated antigens and serum antibody biomarkers. SEREX is based on immunoscreening phage cDNA libraries generated from tumor tissues using patient sera, followed by sequence-based identification of immunoreactive cDNA clones. Since this method enables large-scale and unbiased screening of antigens recognized by the host immune system, it has proven useful for identifying clinically relevant tumor-associated antigens. Using SEREX-based approaches, the present group and others have identified multiple antibody biomarkers in gastrointestinal cancers. In the authors’ previous studies, for example, SKI proto-oncogene protein and transmembrane p24 trafficking protein 5 (TMED5) were identified in esophageal cancer, and α-enolase (ENO1) and SS nuclear autoantigen 1 (SSNA1) in gastric cancer, and the clinical significance of their corresponding serum autoantibodies was subsequently evaluated [9–11]. These findings support the notion that humoral immune responses against tumor-associated antigens may reflect tumor biology and may provide useful information for diagnosis and prognosis.

In the present study, specific focus was placed on CT antigens as a source of candidate biomarkers. Candidate CT antigens were independently screened by analyzing publicly available transcriptomic datasets, and POTEE was selected as a promising target. POTEE belongs to the POTE family of proteins, which are known to show restricted expression in normal tissues and aberrant expression in malignant tissues, suggesting potential relevance to cancer-associated immune responses. On the basis of this rationale, the ability of POTEE to induce a measurable humoral immune response in patients with gastrointestinal cancers was investigated.

To address this question, serum anti-POTEE antibodies (s-POTEE-Abs) were measured in patients with esophageal squamous cell carcinoma, gastric adenocarcinoma, and colorectal adenocarcinoma, and their clinical significance as serological biomarkers was assessed by comparing their diagnostic performance with that of the established marker serum anti-p53 antibodies (s-p53-Abs) using the same serum samples. The association between s-POTEE-Ab positivity and patient prognosis was also evaluated. Through these analyses, the present study aimed to clarify the diagnostic and prognostic value of s-POTEE-Abs in gastrointestinal cancers and to determine whether POTEE-related humoral immunity may serve as a clinically meaningful biomarker platform in these malignancies.

## Materials and methods

### Identification of cancer-testis antigens using commonality as a metric

Candidate cancer-testis antigens were screened using the RNA tissue expression consensus dataset from the Human Protein Atlas (https://www.proteinatlas.org/). Genes were initially selected when they showed significantly higher expression in the testis, ovary, or placenta than in other normal tissues, with a t-test P value < 0.05, a fold change > 20, and zero expression in at least 95% of all other normal tissues. A total of 226 genes met these criteria, including 32 genes previously registered as cancer-testis antigens.

These 226 genes were subsequently evaluated for commonality across tumor transcriptomic datasets. In the present study, commonality was defined as detectable mRNA expression in at least 60% of cases in each relevant cancer dataset. Because an ESCC-specific expression cohort was not available as a separately analyzable dataset in the Human Protein Atlas, the head and neck squamous cell carcinoma (HNSC) dataset was examined only as an exploratory, histologically related surrogate based on the shared squamous histology. The stomach adenocarcinoma (STAD), colon adenocarcinoma (COAD), and rectum adenocarcinoma (READ) datasets were used for gastric and colorectal cancers. Fifteen genes fulfilled the predefined commonality criterion across the analyzed tumor datasets. Among these 15 genes, POTEE was selected for serological investigation because previous evidence had demonstrated T-cell reactivity against POTEE, supporting its potential immunogenicity as a cancer-associated antigen. POTEE also showed broad expression commonality across the tumor datasets examined. The HNSC analysis was regarded as supportive and hypothesis-generating and was not interpreted as direct evidence for ESCC.

In addition, overall survival according to POTEE mRNA expression level was evaluated using reference datasets from the Human Protein Atlas derived from tumor tissues collected at diagnosis. For the exploratory analysis of squamous cell carcinoma, the HNSC dataset was used because an ESCC-specific survival cohort was not separately available in the Human Protein Atlas. Given the substantial biological differences between HNSC and ESCC, the HNSC results were interpreted cautiously and only as supportive, hypothesis-generating evidence. Gastric adenocarcinoma and colorectal adenocarcinoma were analyzed using the STAD and COAD datasets, respectively.

### Collection of serum samples

All serum samples were collected before the initiation of any treatment.

Of the 121 patients with ESCC, 72 underwent surgical resection, whereas 49 received nonsurgical treatment, including chemotherapy and/or radiotherapy. Treatment modality was selected through routine clinical decision-making based on tumor characteristics, operability, comorbidities, multidisciplinary assessment, and patient preference. Pre-treatment serum samples were collected at the Toho University Omori Medical Center between June 2010 and February 2016. All patients were followed for at least five years after treatment. Clinico-pathological data and survival outcomes were retrospectively obtained from medical records.

A total of 62 serum samples were collected from patients with gastric adenocarcinoma, including 43 men and 19 women, while a total of 158 serum samples were collected from patients with colorectal adenocarcinoma, including 98 men and 60 women. All patients underwent surgical resection, and pretreatment serum samples were collected at Toho University Omori Medical Center between May 2010 and May 2013. All patients were followed for at least five years after surgery. Clinicopathological data and survival outcomes were retrospectively collected.

Serum samples from 96 healthy donors were obtained by selective use of residual samples from Port Square Kashiwado Clinic.

No formal a priori sample size calculation was performed because this was a retrospective exploratory study using pretreatment serum samples available during the study period. The sample size of each cancer cohort was therefore determined by the number of available serum samples that could be analyzed.

### Preparation and purification of antigenic POTEE and p53 proteins

Full-length cDNA clones of POTEE and TP53 were purchased from GenScript (Piscataway, NJ, USA) and subcloned into the prokaryotic expression vector pGEX-4T-1 (Cytiva, Pittsburgh, PA, USA). Competent *Escherichia coli* BL21 cells (Nippon Gene, Tokyo, Japan) were transformed with pGEX-4T-1, pGEX-4T-1-POTEE, or pGEX-4T-1-p53 and cultured in 200 mL of Luria broth containing 0.1 mM isopropyl β-D-1-thiogalactopyranoside (IPTG; Wako Pure Chemical Industries, Osaka, Japan) for 3 h.

The bacterial cells were lysed by sonication in BugBuster Protein Extraction Reagent (Merck Millipore, Darmstadt, Germany), and GST, GST-POTEE, and GST-p53 proteins were purified by affinity chromatography using glutathione-Sepharose columns (Cytiva), as previously described [12].

### Measurement of s-POTEE-Ab and s-p53-Ab levels

All study participants provided written informed consent for future use of their blood samples for research purposes. The protocol for the present study was approved by the Ethics Committee of the Toho University (approval nos. T2024-2081, Ota-ku, Tokyo, Japan), and Port Square Kashiwado Clinic, Kashiwado Memorial Foundation (approval no. 2012-001). Patients provided written informed consent before enrolment. The study was registered in the UMIN Clinical Trials Registry (UMIN000014530; registration date: July 11, 2014), and was conducted in accordance with The Declaration of Helsinki and the Japanese Ethical Guidelines for Clinical Research.

Before treatment, 5 mL blood samples were collected, centrifuged at 3,000 × g for 10 min, and the sera were stored at − 80 °C until use. Serum levels of anti-POTEE antibodies (s-POTEE-Abs) and anti-p53 antibodies (s-p53-Abs) were measured by amplified luminescent proximity homogeneous assay-linked immunosorbent assay (AlphaLISA) using 384-well white opaque OptiPlate™ microplates (Revvity, Waltham, MA, USA). Briefly, 2.5 µL of 1:100-diluted serum was mixed with 2.5 µL of AlphaLISA buffer containing 25 mM HEPES (pH 7.4), 0.1% casein, 0.5% Triton X-100, 1 mg/mL dextran-500, and 0.05% Proclin-300, together with GST, GST-POTEE, or GST-p53 protein (10 µg/mL), according to the manufacturer’s instructions and as previously described [10–16]. After incubation at room temperature for 6–8 h, 2.5 µL each of anti-human IgG-conjugated acceptor beads and glutathione-conjugated donor beads (40 µg/mL; Revvity) were added, followed by incubation in the dark at room temperature. Chemiluminescence was measured using an EnSpire Alpha microplate reader (Revvity). Specific reactivity was defined as the Alpha photon counts for each GST-fusion protein minus those for GST alone.

### Comparison of clinico-pathological parameters in treated gastrointestinal cancer patients

Clinicopathological variables were dichotomized and compared with serum biomarker status in patients with gastrointestinal cancers. Specifically, patients were categorized according to sex (male vs. female), age (≤ 64 vs. ≥65 years), tumor depth (T1–2 vs. T3–4), lymph node metastasis (negative vs. positive), and TNM stage group. Associations between clinico-pathological parameters and serum biomarker positivity, including s-POTEE-Ab, s-p53-Ab, SCC-Ag, and CEA, were evaluated using Fisher’s exact test.

**Fig. 1:**
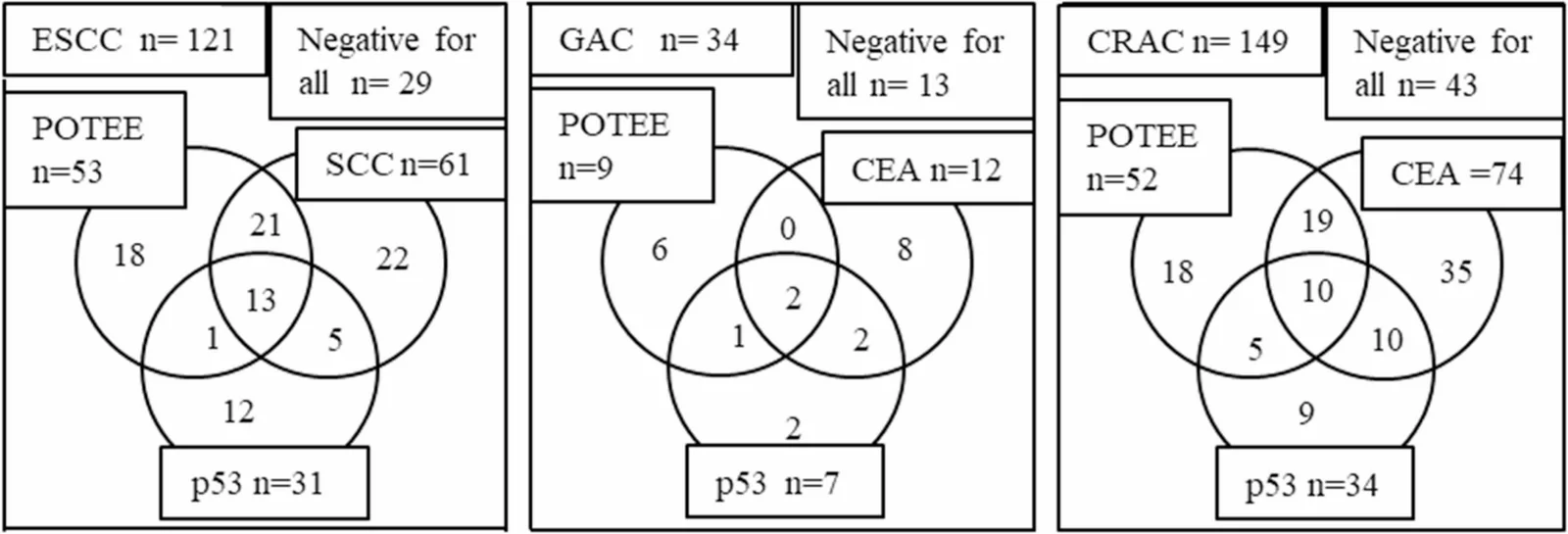
Overlap of positive samples for s-POTEE-Abs, s-p53-Abs, SCC, and CEA in patients with gastrointestinal cancers. The cutoff value for s-POTEE-Ab was defined as the mean level in healthy donors plus 2 standard deviations, whereas the cutoff values for the other markers were based on predefined clinical criteria. The numbers in the figure indicate the numbers of samples positive for single, double, or triple combinations of these markers. The analyses were restricted to patients with complete data for all markers shown: 121 patients with ESCC, 34 patients with GAC, and 149 patients with CRAC. CEA and SCC-Ag were measured as part of routine clinical care and were unavailable in some patients because these tests had not been ordered by the treating physicians. ESCC, esophageal squamous cell carcinoma; GAC, gastric adenocarcinoma; CRAC, colorectal adenocarcinoma; SCC-Ag, squamous cell carcinoma antigen; CEA, carcinoembryonic antigen

**Fig. 2:**
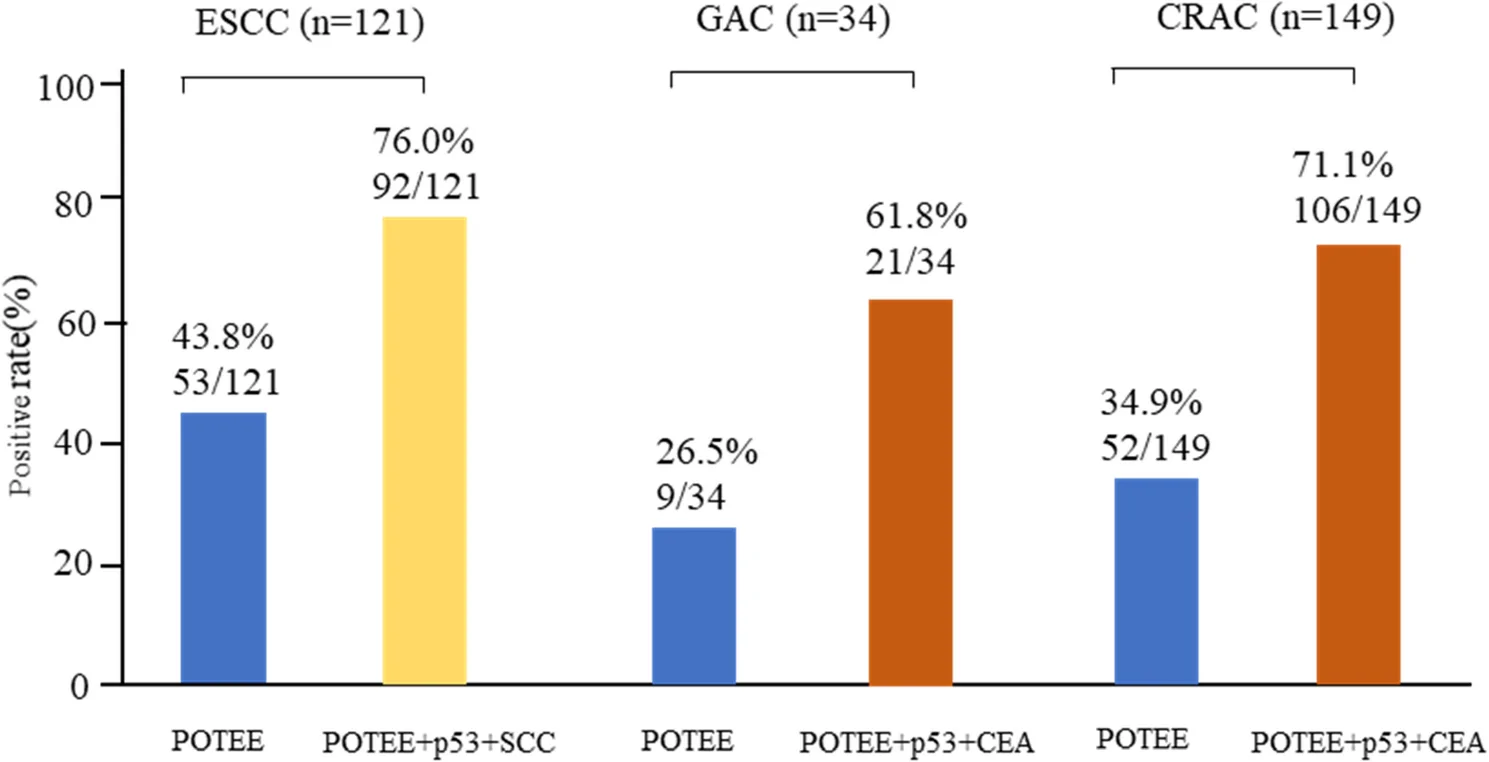
Positivity rates of s-POTEE-Abs alone and combined serum markers. The cutoff value for s-POTEE-Ab was defined as the mean value in healthy donors plus 2 standard deviations. Combined positivity was defined as positivity for at least one of the indicated markers. Combined-positivity analyses were restricted to patients with complete measurements of all markers included in each panel. Complete data were available for all 121 patients with ESCC, 34 of 62 patients with GAC, and 149 of 158 patients with CRAC. The reduced sample sizes in the GAC and CRAC cohorts were attributable to unavailable CEA measurements in routine clinical practice. The patient positivity rates increased from 43.8% (53/121) to 76.0% (92/121) in ESCC with s-POTEE-Ab, s-p53-Ab, and SCC-Ag; from 26.5% (9/34) to 61.8% (21/34) in GAC with s-POTEE-Ab, s-p53-Ab, and CEA; and from 34.9% (52/149) to 71.1% (106/149) in CRAC with s-POTEE-Ab, s-p53-Ab, and CEA. Because SCC-Ag and CEA were not measured in healthy donors, these findings indicate complementary positivity among patients and do not constitute a formal comparison of diagnostic accuracy

Serum s-POTEE-Ab and s-p53-Ab levels were available for all patients and were used for the antibody-level comparisons and ROC analyses. Analyses of individual serum markers in Tables 1 and 2 used the available data for each marker; therefore, the number of evaluable patients could differ by marker. In contrast, the marker-overlap and combined-positivity analyses in Figs. 1 and 2 required complete measurements of all markers included in each panel and were restricted to 34 patients with GAC and 149 patients with CRAC. Cox proportional hazards analyses were conducted using complete cases with available survival and covariate data for the variables included in each model.

**Table 1:** Clinicopathological significance of positive three biomarkers (s-POTEE-Abs, CEA, s-p53-Abs) in gastric adenocarcinoma

	No.of patients	s-POTEE-Abs		CEA		s-p53-Abs	
		> 11241^b^	*P* value^a^	> 5.0 ng/mL^c^	*P* value^a^	> 1.3U/mlc	*P* value^a^
*n* = 62							
Gender							
Female	19	11	0.411	3	0.119	1	0.422
Male	43	19		13		6	
Age (years old)							
≤ 65	26	14	0.607	8	0.559	5	0.119
> 65	36	16		8		2	
Tumor depth							
T1,T2	30	15	1	5	0.15	5	0.231
T3, T4	32	15		11		2	
Nodal status							
Negative	30	15	1	4	0.042	2	0.427
Positive	32	15		12		5	
Stage							
I/II	38	18	1	7	0.758	3	0.415
III/IV	24	12		9		4	

**Table 2:** Clinicopathological significance of positive three biomarkers (s-POTEE-Abs, CEA, s-p53-Abs) in colorectal adenocarcinoma

		s-POTEE-Abs		CEA		s-p53-Abs	
		> 7823 ^b^	*P* value^a^	> 5.0 ng/mL	*P* value^a^	> 1.3U/ml	*P* value^a^
*n* = 158							
Gender							
Female	60	38	0.268	27	0.729	13	1
Male	98	71		49		21	
Age (years old)							
≤ 65	75	48	0.23	42	0.079	18	0.562
> 65	83	61		34		16	
Tumor depth							
T1, T2	52	34	0.583	15	< 0.01	6	< 0.05
T3, T4	106	75		61		28	
Nodal status							
Negative	101	70	1	40	0.586	21	0.841
Positive	57	39		36		13	
Stage							
I/II	95	66	1	36	< 0.01	19	0.693
III/IV	63	43		40		15	

^a^Fisher’s exact probability test. ^b^ Optimal cut-off values for serum antibody levels that affect overall survival were identified with X-tile version 3.6.1 software (Yale University, New Haven, CT). ^c^Using cut-off values in clinical practice. s-POTEE-Abs, serum anti-POTEE antibodies; CEA, Carcinoembryonic Antigen; s-p53-Abs, serum anti-p53 antibodies. The cohort size shown in the table represents the full study cohort. Analyses for each biomarker were based on patients with available measurements for that marker; therefore, evaluable denominators could differ by biomarker. Figures 1 and 2 were restricted to patients with complete data for all markers in the corresponding panel

For clinico-pathological analysis, the cutoff values for s-p53-Ab, SCC-Ag, and CEA were set at 1.3 U/mL, 1.5 ng/mL, and 5.0 ng/mL [17–19], following the manufacturer’s instruction. For prognostic analysis, the cutoff levels of serum markers were determined using X-tile software (Yale University, New Haven, CT, USA), which identifies the optimal threshold based on the log-rank test.

### Statistical analysis

Associations between categorical variables were analyzed using Fisher’s exact test. Continuous variables between healthy donors and patients with gastrointestinal cancers were compared using the Mann–Whitney U test. Receiver operating characteristic (ROC) curve analysis was performed to evaluate the diagnostic performance of s-POTEE-Ab and s-p53-Ab and to determine optimal cutoff values using the Youden index. Sensitivity, specificity, positive predictive value (PPV), negative predictive value (NPV), and their 95% confidence intervals (CIs) were calculated at the selected cutoff values. Because SCC-Ag and CEA were not measured in the healthy donor cohort, formal diagnostic accuracy analyses incorporating these markers could not be validly performed. Therefore, the combined diagnostic analysis was restricted to s-POTEE-Ab and s-p53-Ab, both of which were measured in all patients and healthy donors. For each cancer type, a multivariable logistic regression model was constructed using the continuous levels of s-POTEE-Ab and s-p53-Ab as explanatory variables and cancer status as the dependent variable. The predicted probabilities derived from these models were used for ROC analysis. AUCs for single markers and the combined models were compared using DeLong’s test for correlated ROC curves. PPV and NPV were calculated within the present case–control study population and were not interpreted as estimates for a population-based screening setting. All diagnostic analyses were performed using EZR software [20].

To determine optimal cutoff levels for survival analysis in treated gastrointestinal cancer patients, X-tile software (Yale University, New Haven, CT, USA) [21] was used. Overall survival curves were generated using the Kaplan-Meier method and compared using the log-rank test. Variables associated with overall survival in the univariate analyses were entered into the multivariable Cox proportional hazards models. The ESCC model included age, stage, treatment modality (nonsurgical treatment vs. surgery), s-POTEE-Ab status, SCC-Ag status, and s-p53-Ab status. Treatment modality was included irrespective of its univariate P value because of its potential confounding effect. The GAC model included tumor depth, lymph node status, and stage. The CRAC model included tumor depth, lymph node status, stage, s-POTEE-Ab status, and CEA status. Cox analyses were performed using complete cases with available survival and covariate data. All other statistical analyses were performed using EZR software [20]. A two-sided P value < 0.05 was considered statistically significant.

## Results

### Identification of POTEE as a candidate cancer-testis antigen

Using the predefined normal-tissue expression criteria, 226 candidate genes were identified from the Human Protein Atlas RNA tissue expression consensus dataset (Supplementary Table S1). Of these, 32 had previously been registered as cancer-testis antigens. The 226 genes were subsequently assessed for tumor-expression commonality, defined as detectable expression in at least 60% of cases in each analyzed tumor dataset. Fifteen genes met this criterion across the HNSC, STAD, COAD, and READ datasets. Among these candidates, POTEE was selected as the primary subject for serological investigation because it showed broad tumor-expression commonality and had previously been reported to elicit T-cell reactivity, providing independent evidence of its potential immunogenicity as a cancer-associated antigen (Supplementary Table S2).

### Comparison of s-POTEE-Ab and s-p53-Ab levels between healthy donors and patients with gastrointestinal cancers

**Fig. 3:**
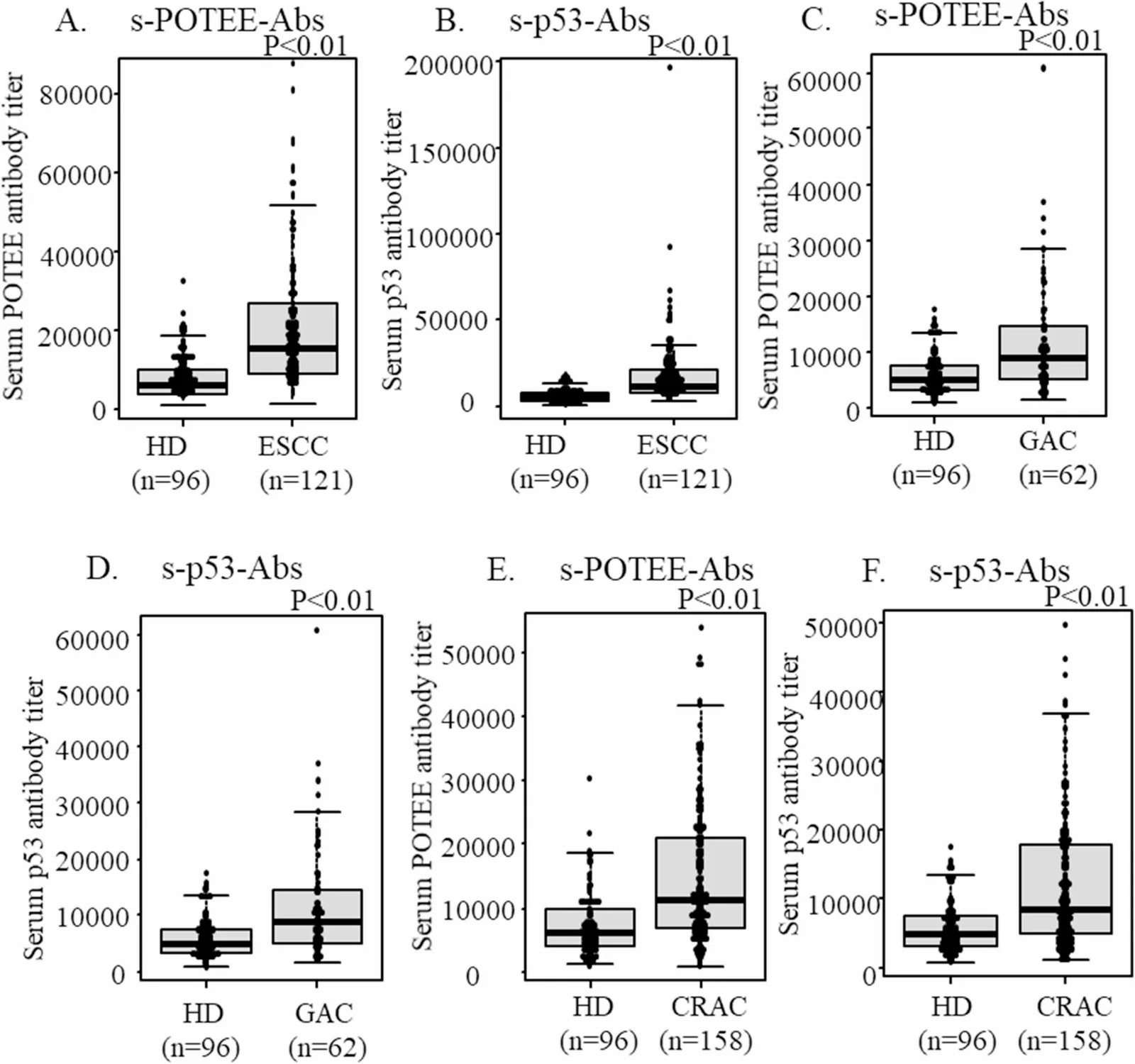
Comparison of serum s-POTEE-Ab and s-p53-Ab levels between healthy donors and surgically treated patients with gastrointestinal cancers. The levels of (**A**) s-POTEE-Abs and (**B**) s-p53-Abs in patients with esophageal squamous cell carcinoma (ESCC) and healthy donors were examined using the AlphaLISA assay. The levels of (**C**) s-POTEE-Abs and (**D**) s-p53-Abs in patients with gastric adenocarcinoma (GAC) and healthy donors are also shown. The levels of (**E**) s-POTEE-Abs and (**F**) s-p53-Abs in patients with colorectal adenocarcinoma (CRAC) and healthy donors are likewise shown. Data are presented as box-and-whisker plots. The boxes represent the 25th, 50th, and 75th percentiles, and the whiskers indicate the 10th and 90th percentiles. Statistical analysis was performed using the Mann–Whitney U test. *P* < 0.01

Serum s-POTEE-Abs and s-p53-Abs levels were measured by the AlphaLISA assay using GST-POTEE and GST-p53 as antigens. The levels of both s-POTEE-Abs and s-p53-Abs were significantly higher in patients with gastrointestinal cancers than in healthy donors (Fig. 3A–F, *P* < 0.01).

**Fig. 4:**
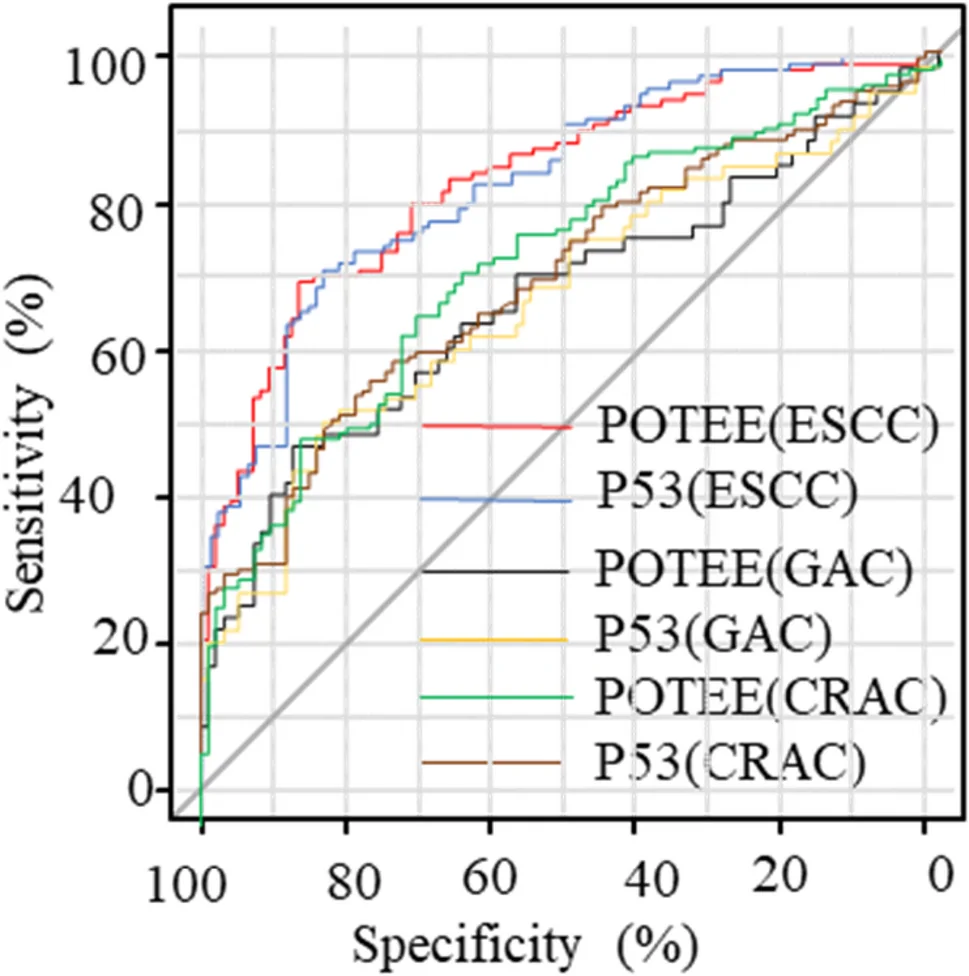
The ROC curve analysis yielded the following results for each carcinoma: For esophageal squamous cell carcinoma (ESCC), the area under the ROC curve (AUC) was 0.832 (95% CI: 0.779–0.885) for s-POTEE-Abs and 0.829 (95% CI: 0.776–0.882) for s-p53-Abs (*n* = 121 cases vs. 96 controls). In gastric adenocarcinoma (GAC), the AUC was 0.680 (95% CI: 0.590–0.771) for s-POTEE-Abs and 0.698 (95% CI: 0.611–0.785) for s-p53-Abs (*n* = 62 cases vs. 96 controls). For colorectal adenocarcinoma (CRAC), the AUC reached 0.735 (95% CI: 0.674–0.797) for s-POTEE-Abs and 0.706 (95% CI: 0.643–0.769) for s-p53-Abs(*n* = 158 cases vs. 96 controls)

ROC curve analysis was performed to evaluate the diagnostic performance of s-POTEE-Ab and s-p53-Ab in each carcinoma. In ESCC, the AUCs for s-POTEE-Ab and s-p53-Ab were 0.832 (95% CI, 0.779–0.885) and 0.829 (95% CI, 0.776–0.882), respectively (121 cases vs. 96 controls), with no significant difference by DeLong’s test (*P* = 0.765). At the Youden index-based cutoffs of 11,800 and 8,605, the sensitivities were 69.4% and 71.1%, and the specificities were 86.5% and 83.3%, respectively. In GAC, the corresponding AUCs were 0.680 (95% CI, 0.590–0.771) and 0.698 (95% CI, 0.611–0.785), respectively (62 cases vs. 96 controls), with no significant difference between the two markers (*P* = 0.172). At cutoffs of 12,741 and 8,546, the sensitivities were 48.4% and 53.2%, and the specificities were 87.5% and 83.3%, respectively. In CRAC, the AUC for s-POTEE-Ab was significantly higher than that for s-p53-Ab [0.735 (95% CI, 0.674–0.797) vs. 0.706 (95% CI, 0.643–0.769); 158 cases vs. 96 controls; *P* = 0.008 by DeLong’s test]. At cutoffs of 8,317 and 8,025, the sensitivities were 66.5% and 55.1%, and the specificities were 70.8% and 79.2%, respectively (Fig. 4; Table 3).

**Table 3:** Diagnostic performance of s-POTEE-Ab, s-p53-Ab, and their combined logistic regression models

Cancer type	Marker/model	Cutoff	Sensitivity, % (95% CI)	Specificity, % (95% CI)	PPV, % (95% CI)	NPV, % (95% CI)	AUC (95% CI)	*P* vs. combined model
ESCC	s-POTEE-Ab	11,800	69.4 (60.4–77.5)	86.5 (78.0–92.6)	86.6 (78.2–92.7)	69.2 (60.1–77.3)	0.832 (0.779–0.885)	0.956
ESCC	s-p53-Ab	8,605	71.1 (62.1–79.0)	83.3 (74.4–90.2)	84.3 (75.8–90.8)	69.6 (60.3–77.8)	0.829 (0.776–0.882)	0.555
ESCC	Combined model	0.545	71.1 (62.1–79.0)	83.3 (74.4–90.2)	84.3 (75.8–90.8)	69.6 (60.3–77.8)	0.831 (0.779–0.884)	Reference
GAC	s-POTEE-Ab	12,741	48.4 (35.5–61.4)	87.5 (79.2–93.4)	71.4 (55.4–84.3)	72.4 (63.3–80.3)	0.680 (0.590–0.771)	0.215
GAC	s-p53-Ab	8,546	53.2 (40.1–66.0)	83.3 (74.4–90.2)	67.3 (52.5–80.1)	73.4 (64.1–81.4)	0.698 (0.611–0.785)	0.475
GAC	Combined model	0.413	53.2 (40.1–66.0)	84.4 (75.5–91.0)	68.8 (53.7–81.3)	73.6 (64.4–81.6)	0.702 (0.616–0.788)	Reference
CRAC	s-POTEE-Ab	8,317	66.5 (58.5–73.8)	70.8 (60.7–79.7)	78.9 (71.0–85.5)	56.2 (46.9–65.2)	0.735 (0.674–0.797)	0.332
CRAC	s-p53-Ab	8,025	55.1 (47.0–63.0)	79.2 (69.7–86.8)	81.3 (72.6–88.2)	51.7 (43.3–60.0)	0.706 (0.643–0.769)	0.0039
CRAC	Combined model	0.552	69.0 (61.2–76.1)	68.8 (58.5–77.8)	78.4 (70.6–84.9)	57.4 (47.8–66.6)	0.733 (0.672–0.795)	Reference

Cutoff values were determined using the Youden index. Combined models were constructed using multivariable logistic regression incorporating continuous s-POTEE-Ab and s-p53-Ab levels. AUCs were compared using the paired DeLong test. PPV and NPV were calculated based on the proportions of patients and healthy donors included in the present case-control dataset and should not be interpreted as population-based predictive values

Combined logistic regression models incorporating the continuous levels of s-POTEE-Ab and s-p53-Ab yielded AUCs of 0.831 (95% CI, 0.779–0.884) in ESCC, 0.702 (95% CI, 0.616–0.788) in GAC, and 0.733 (95% CI, 0.672–0.795) in CRAC. In ESCC, the combined model showed a sensitivity of 71.1%, specificity of 83.3%, PPV of 84.3%, and NPV of 69.6% at a predicted-probability cutoff of 0.545. Its AUC did not differ significantly from that of s-POTEE-Ab alone (*P* = 0.956) or s-p53-Ab alone (*P* = 0.555). In GAC, the combined model showed a sensitivity of 53.2%, specificity of 84.4%, PPV of 68.8%, and NPV of 73.6% at a cutoff of 0.413. Its AUC did not differ significantly from that of s-POTEE-Ab alone (*P* = 0.215) or s-p53-Ab alone (*P* = 0.475). In CRAC, the combined model showed a sensitivity of 69.0%, specificity of 68.8%, PPV of 78.4%, and NPV of 57.4% at a cutoff of 0.552. Its AUC was significantly higher than that of s-p53-Ab alone (*P* = 0.0039) but was not significantly different from that of s-POTEE-Ab alone (*P* = 0.332). Thus, combining the two antibodies did not improve discrimination beyond the better-performing single marker in any of the three cancer types (Table 3). The higher positivity rates observed when s-POTEE-Ab, s-p53-Ab, SCC-Ag, and CEA were considered together reflected partly non-overlapping positivity patterns among patients and should not be interpreted as evidence of synergistic effects, improved diagnostic accuracy, or superiority over individual markers.

Serum s-POTEE-Ab and s-p53-Ab measurements were available for all 62 patients with GAC and all 158 patients with CRAC and were used for the antibody-level and ROC analyses. For analyses of individual conventional markers in Tables 1 and 2, all available marker-specific data were used. However, the marker-overlap and combined-positivity analyses required complete measurements of every marker in each panel and were therefore restricted to 34 patients with GAC and 149 patients with CRAC because CEA had not been measured in the remaining patients during routine clinical care. Within these complete-case subsets, the positivity rate of s-POTEE-Ab was 26.5% (9/34) in GAC and 34.9% (52/149) in CRAC; the corresponding rate in ESCC was 43.8% (53/121). s-POTEE-Ab positivity showed limited overlap with s-p53-Ab, SCC-Ag, and CEA positivity, indicating distinct positivity patterns among these serum markers (Fig. 1).

A flow diagram showing the numbers of patients included in each diagnostic and marker-overlap analysis is provided in Supplementary Figure S2.

### Association between s-POTEE-Ab positivity and clinico-pathological parameters

Associations between biomarker positivity and clinico-pathological variables were evaluated using cutoff values determined by X-tile analysis and Fisher’s exact probability test (Tables 1, 2 and 4). In ESCC, SCC-Ag positivity was significantly associated with greater tumor depth (*P* < 0.05), positive nodal status (*P* < 0.01), and advanced stage (*P* < 0.01). In contrast, neither s-POTEE-Ab, nor s-p53-Ab positivity was significantly associated with clinicopathological variables, although s-p53-Ab positivity tended to be associated with advanced stage (*P* = 0.0532).

**Table 4:** Clinicopathological significance of positive three biomarkers (s-POTEE-Abs, SCC, s-p53-Abs) in esophageal squamous cell carcinoma

	No.of patients	s-POTEE-Abs		SCC		s-p53-Abs	
		> 33,350^b^	*P* value^a^	> 1.5 ng/mL^c^	*P* value^a^	> 1.3U/mlc	*P* value^a^
(*n* = 121)							
Gender							
Female	18	0	0.0727	9	1	6	0.397
Male	103	19		52		25	
Age (years old)							
≤ 65	46	8	0.798	24	0.852	14	0.394
> 65	75	11		37		17	
Tumor depth							
T1, T2	48	8	0.804	18	< 0.05	9	0.203
T3, T4	73	11		43		22	
Nodal status							
Negative	42	5	0.446	14	< 0.01	8	0.278
Positive	79	14		47		23	
Stage							
I/II	46	5	0.31	14	< 0.01	7	0.0532
III/IV	75	14		47		24	

^a^Fisher’s exact probability test. ^b^ Optimal cut-off values for serum antibody levels that affect overall survival were identified with X-tile version 3.6.1 software (Yale University, New Haven, CT). ^c^Using cut-off values in clinical practice. s-POTEE-Abs, serum anti-POTEE antibodies; SCC, Squamous Cell Carcinoma Antigen; s-p53-Abs, serum anti-p53 antibodies

In GAC, CEA positivity was significantly associated with nodal positivity (*P* < 0.05), whereas neither s-POTEE-Ab nor s-p53-Ab positivity showed significant associations with clinicopathological parameters. In CRAC, CEA positivity was significantly associated with deeper tumor invasion (*P* < 0.01) and advanced stage (*P* < 0.01), and s-p53-Ab positivity was significantly associated with greater tumor depth (*P* < 0.05). No significant associations were observed between s-POTEE-Ab positivity and clinico-pathological parameters in any of the three cancer types.

### Univariate and multivariate analysis of prognostic factors

Univariate and multivariate analyses were performed to evaluate the prognostic significance of clinicopathological variables, including autoantibody status, for overall survival in patients with ESCC, GAC, and CRAC (Tables 5, 6 and 7).

**Table 5:** Univariate and multivariate analysis of the risk factors associated with the overall survival of 121 patients with esophageal squamous cell carcinoma

Variable	Univariate analysis		Multivariate analysis	
	*P*-value^a^	Hazard ratio	95% CI	*P*-value^b^
Gender	0.301			
Male/Female				
Age (yeras old)	0.038	1.029	0.999–1.060	0.057
> 65/≤65				
Tumor depth	< 0.01			
T1,T2/T3, T4				
Nodal status	< 0.01			
Negative/Positive				
Stage	< 0.01	2.128	1.576–2.875	< 0.01
I, II/III, IV				
Treatment modality	0.593	1.181	0.692–2.014	0.542
Nonsurgical treatment vs. surgery				
s-POTEE-Abs	0.034	1.169	0.564–2.422	0.675
Negative/Positive				
SCC	0.042	1.184	0.692–2.026	0.538
Negative/Positive				
s-p53-Abs	0.011	1.746	0.944–3.230	0.076
Negative/Positive				

^a^Log-rank test. ^b^Cox proportional hazard model. *P* < 0.05 was considered to indicate a statistically significant difference. s-POTEE-Abs, serum anti-POTEE antibodies; SCC, Squamous Cell Carcinoma Antigen; s-p53-Abs, serum anti-p53 antibodies

**Table 6:** Univariate and multivariate analyses of factors associated with overall survival in the gastric adenocarcinoma cohort (*n* = 62)

Variable	Univariate analysis		Multivariate analysis	
	*P*-value^a^	Hazard ratio	95% CI	*P*-value^b^
Gender	0.528	0.744	0.296–1.872	0.530
Male/Female				
Age (yeras old)	0.241	1.017	0.974–1.062	0.437
> 65/≤65				
Tumor depth	< 0.01	3.832	1.571–9.347	< 0.01
T1, T2/T3, T4				
Nodal status	< 0.01	5.537	2.062–14.87	< 0.01
Negative/Positive				
Stage	< 0.01	3.932	1.722–8.980	< 0.01
I, II/III, IV				
s-POTEE-Abs	0.323	1.486	0.673–3.281	0.327
Negative/Positive				
CEA	0.81	1.113	0.464–2.671	0.810
Negative/Positive				
s-p53-Abs	0.779	0.802	0.171–3.761	0.779
Negative/Positive				

^a^Log-rank test. ^b^Cox proportional hazard model. *P* < 0.05 was considered to indicate a statistically significant difference. s-POTEE-Abs, serum anti-POTEE antibodies; CEA, Carcinoembryonic Antigen; s-p53-Abs, serum anti-p53 antibodies Multivariable Cox proportional hazards analyses were performed using complete cases with available survival and covariate data for all variables included in the model

**Table 7:** Univariate and multivariate analyses of factors associated with overall survival in the colorectal adenocarcinoma cohort (*n* = 158)

Variable		Univariate analysis		Multivariate analysis	
		*P*-value^a^	Hazard ratio	95% CI	*P*-value^b^
Gender		0.462	0.720	0.388–1.334	0.296
Male/Female					
Age (yeras old)	0.388		1.266	0.388–1.334	0.432
> 65/≤65					
Tumor depth	< 0.01		8.769	2.718–28.3 0	< 0.01
T1, T2/T3, T4					
Nodal status	< 0.01		5.359	2.883–9.962	< 0.01
Negative/Positive					
Stage		< 0.01	7.712	3.817–15.58	< 0.01
I, II/III, IV					
s-POTEE-Abs	0.027		2.308	1.076–4.949	< 0.05
Negative/Positive					
CEA		0.037	1.844	1.027–3.313	< 0.05
Negative/Positive					
s-p53-Abs		0.976	0.989	0.487–2.007	0.976
Negative/Positive					

^a^Log-rank test. ^b^Cox proportional hazard model. *P* < 0.05 was considered to indicate a statistically significant difference. s-POTEE-Abs, serum anti-POTEE antibodies; CEA, Carcinoembryonic Antigen; s-p53-Abs, serum anti-p53 antibodies Multivariable Cox proportional hazards analyses were performed using complete cases with available survival and covariate data for all variables included in the model

In ESCC, univariate analysis showed that age, tumor depth, nodal status, stage, s-POTEE-Ab positivity, SCC-Ag positivity, and s-p53-Ab positivity were significantly associated with overall survival, whereas sex and treatment modality were not. In the multivariable Cox model incorporating age, stage, treatment modality, s-POTEE-Ab, SCC-Ag, and s-p53-Ab, stage remained independently associated with overall survival (HR, 2.128; 95% CI, 1.576–2.875; *P* < 0.001). Treatment modality was not significantly associated with overall survival (HR, 1.181; 95% CI, 0.692–2.014; *P* = 0.542). After adjustment for stage and treatment modality, s-POTEE-Ab positivity was not independently associated with overall survival in the ESCC cohort (HR, 1.169; 95% CI, 0.564–2.422; *P* = 0.675).

In GAC, tumor depth, nodal status, and stage were significantly associated with overall survival in both univariate and multivariate analyses, whereas sex, age, s-POTEE-Ab positivity, CEA positivity, and s-p53-Ab positivity were not significant prognostic factors.

In CRAC, tumor depth, nodal status, stage, s-POTEE-Ab positivity, and CEA positivity were significantly associated with overall survival in both univariate and multivariate analyses, whereas sex, age, and s-p53-Ab positivity were not. Thus, s-POTEE-Ab positivity remained an independent prognostic factor in CRAC but not in GAC; in ESCC, its significant univariate association with overall survival was not retained after adjustment for stage and treatment modality.

### Association between POTEE mRNA expression and prognosis in public datasets

In an exploratory analysis of public datasets, high POTEE mRNA expression was significantly associated with poorer overall survival in HNSC. Because HNSC was examined only as a histologically related surrogate and differs biologically from ESCC, this finding should not be interpreted as direct evidence of a prognostic association in ESCC. In STAD and COAD, high POTEE expression showed nonsignificant trends toward poorer overall survival (Supplementary Figure S1).

### Kaplan–Meier survival analysis according to serum biomarker status

**Fig. 5:**
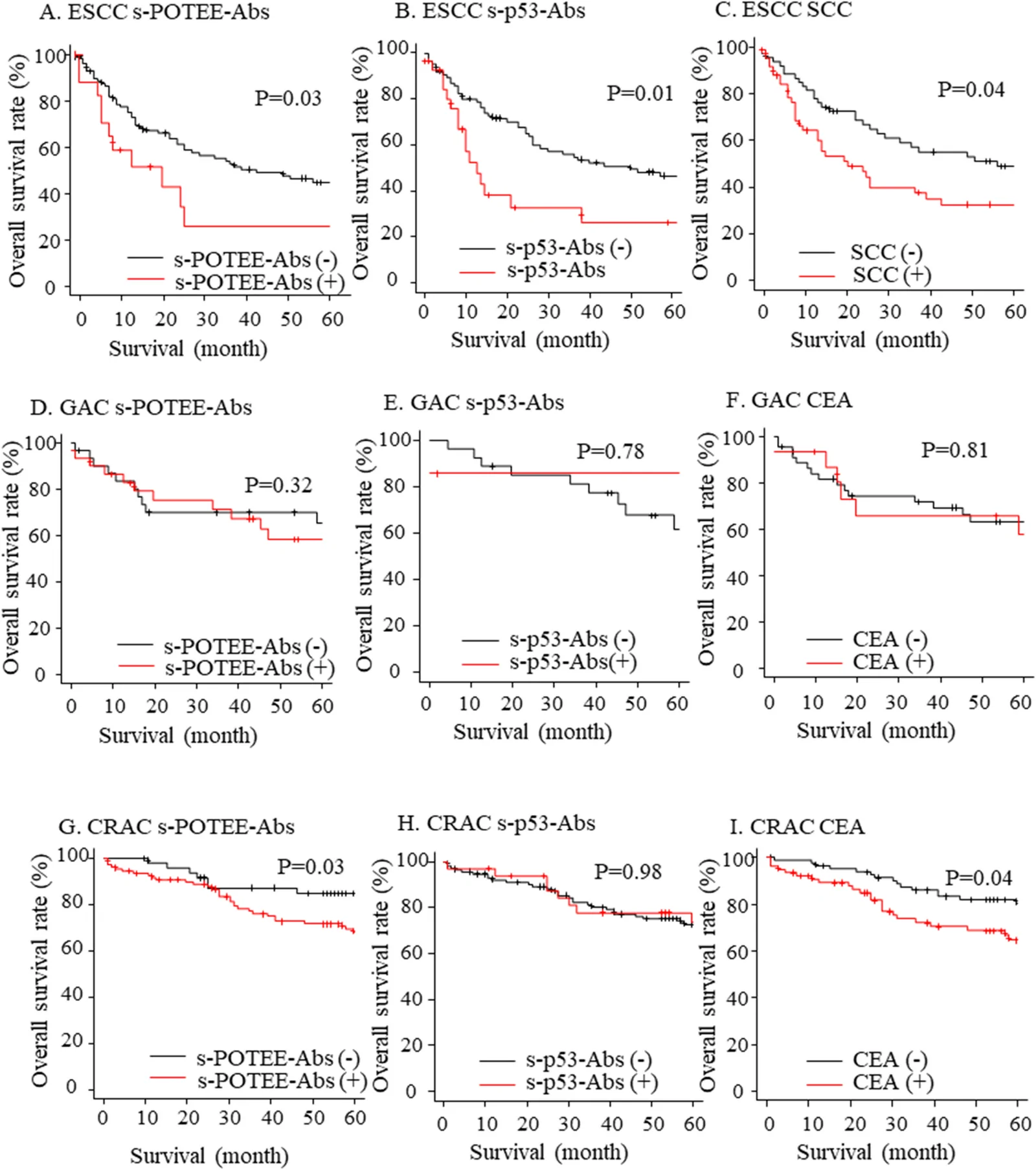
Comparison of overall survival between the positive and negative groups according to s-POTEE-Abs, s-p53-Abs, SCC, and CEA in gastrointestinal cancers. Kaplan–Meier plots for overall survival in treated esophageal squamous cell carcinoma (ESCC) are shown in (A), (B), and (C). Kaplan–Meier plots for overall survival in surgically treated gastric adenocarcinoma (GAC) are shown in (D), (E), and (F). Kaplan–Meier plots for overall survival in surgically treated colorectal adenocarcinoma (CRAC) are shown in (G), (H), and (I). Patients were divided into positive and negative groups according to the serum levels of s-POTEE-Abs, s-p53-Abs, SCC, and CEA. The cut-off values for s-POTEE-Abs were determined by X-tile analysis as follows: ESCC, 33,350 (A); GAC, 11,241 (D); and CRAC, 7,823 (G). Comparisons according to s-p53-Abs status were based on a cut-off value of 1.3 U/ml in (B), (E), and (H). Comparisons according to SCC status were based on a cut-off value of 1.5 ng/ml in (C). Comparisons according to CEA status were based on a cut-off value of 5.0 ng/ml in (F) and (I)

The prognostic significance of serum biomarkers was further evaluated using Kaplan–Meier survival analysis (Fig. 5). Patients were classified into positive and negative groups according to the cutoff values determined by X-tile analysis.

In ESCC and CRAC, patients positive for s-POTEE-Abs had significantly poorer overall survival than those negative for s-POTEE-Abs. In contrast, no significant difference in overall survival according to s-POTEE-Ab status was observed in GAC (Fig. 5A, D, and G). These findings indicate that the prognostic association of s-POTEE-Ab was not consistent across gastrointestinal cancer types.

Patients with s-p53-Ab levels > 1.3 U/mL were classified as positive. In ESCC, the s-p53-Ab-positive group showed significantly poorer overall survival, whereas no significant survival difference was observed in GAC or CRAC. Patients with SCC-Ag levels > 1.5 ng/mL were classified as positive, and in ESCC, SCC-Ag positivity was significantly associated with poorer overall survival (Fig. 5C). Patients with CEA levels > 5.0 ng/mL were classified as positive. In CRAC, the CEA-positive group showed significantly poorer overall survival, whereas no significant difference was observed in GAC (Fig. 5F and I).

## Discussion

In the present study, POTEE was identified as a candidate CTA through an independent screening strategy based on publicly available transcriptomic datasets, and the clinical significance of s-POTEE-Abs in gastrointestinal cancers was investigated. The principal findings were as follows: first, serum s-POTEE-Ab levels were significantly elevated in patients with ESCC, GAC, and CRAC compared with healthy donors; second, the diagnostic performance of s-POTEE-Ab was comparable to that of s-p53-Ab; third, logistic regression models combining s-POTEE-Ab and s-p53-Ab did not show a clear improvement in AUC over either marker alone; fourth, s-POTEE-Ab positivity showed limited overlap with conventional serum biomarkers and increased patient positivity when assessed together with these markers, although formal diagnostic accuracy could not be evaluated for panels containing SCC-Ag or CEA because these markers were not measured in healthy donors; and fifth, s-POTEE-Ab positivity was significantly associated with poorer overall survival in univariate analyses of ESCC and CRAC, but remained an independent prognostic factor only in CRAC after multivariable adjustment.

CTAs have attracted considerable attention because of their highly restricted expression in normal tissues and aberrant expression in malignant tissues. Their expression in immuno-privileged tissues such as the testis and placenta, is thought to contribute to the induction of tumor-associated immune responses when ectopically expressed in cancer cells. On the basis of this biological background, CTAs have been widely investigated as immunotherapeutic targets and as biomarkers reflecting tumor-associated immune recognition [5, 6]. The present study focused on POTEE as a CTA candidate. POTEE has recently been identified as an immunogenic cancer-associated antigen and a potential immunotherapy target in ovarian cancer [22]. The present study further demonstrated that a humoral immune response against POTEE can be detected in the sera of patients with gastrointestinal cancers. This finding suggests that POTEE is immunogenic in these malignancies and supports its potential relevance as a serological biomarker.

A notable finding of the present study was that the diagnostic performance of s-POTEE-Ab was comparable to that of s-p53-Ab across all three cancer types examined. The combined logistic regression models incorporating both antibody levels yielded AUCs of 0.831 in ESCC, 0.702 in GAC, and 0.733 in CRAC and did not provide a clear improvement over the better-performing single marker in each cancer type. Thus, although s-POTEE-Ab and s-p53-Ab may reflect different aspects of tumor-associated humoral immunity, their combination did not materially improve discrimination in the present cohorts. The limited overlap between s-POTEE-Ab positivity and positivity for s-p53-Ab, SCC-Ag, and CEA indicates that these markers identify partly distinct patient subsets. However, SCC-Ag and CEA were available only for the patient cohorts and were not measured in healthy donors. Consequently, the higher patient positivity rates observed when these markers were considered together cannot be interpreted as synergistic effects, improved sensitivity and specificity, superior AUC, or superiority over individual markers. These results should instead be regarded as evidence of complementary patient detection. Moreover, the absolute AUC values, particularly in GAC, remained modest; therefore, s-POTEE-Ab alone or in combination with s-p53-Ab is unlikely to be sufficient as a standalone diagnostic test. The prognostic utility of multimarker combinations was not evaluated using a prespecified prediction model; consequently, their value for prognostic stratification remains undetermined. Independent validation using prospectively collected controls in whom all candidate markers are measured is required.

The prognostic relevance of s-POTEE-Ab appeared to differ among cancer types. Significant univariate associations with poorer overall survival were observed in ESCC and CRAC, whereas no such association was detected in GAC. In CRAC, the association remained significant after multivariable adjustment. In ESCC, however, it was not retained after adjustment for stage and treatment modality, indicating that the unadjusted association may have been influenced by disease stage and treatment background. Therefore, s-POTEE-Ab should not be interpreted as a uniformly applicable prognostic marker across gastrointestinal cancers. The observed associations raise the possibility that the humoral immune response against POTEE may reflect aggressive tumor behavior, increased tumor burden, enhanced antigen release, or greater immunogenic exposure during tumor progression. Further mechanistic and validation studies are warranted.

The candidate-screening strategy should be interpreted with caution because HNSC was used as an exploratory, histologically related surrogate in the absence of a separately analyzable ESCC-specific cohort in the Human Protein Atlas. Although HNSC and ESCC share squamous histology, they differ substantially in anatomical site, etiological factors, HPV association, and molecular background. Therefore, the HNSC-based screening and survival results cannot be regarded as direct validation in ESCC and should be considered supportive and hypothesis-generating only. Confirmation using an ESCC-specific transcriptomic and survival dataset is required.

Interestingly, despite its diagnostic elevation in GAC, s-POTEE-Ab positivity was not significantly associated with prognosis in this cancer type. Several explanations may account for this result. First, the biological role of POTEE and the host immune response against it may differ among gastrointestinal cancer types. Second, the prognostic impact of serum autoantibodies may be influenced by disease stage distribution, treatment background, and tumor heterogeneity. Third, and importantly, the GAC cohort was relatively small (*n* = 62), and no formal a priori sample size calculation was performed. The limited sample size may have produced imprecise or unstable estimates in subgroup analyses and may have increased the risk of false-negative findings and selection-related bias. Therefore, the absence of significant clinicopathological or prognostic associations in GAC should not be interpreted as definitive evidence of no association. Because the present retrospective cohort could not be expanded and no independent validation cohort was available, the GAC findings should be regarded as preliminary and exploratory and require validation in a larger, prospectively collected, independent cohort.

Moreover, the present findings should be considered in the context of the authors’ previous SEREX-based studies [10–17] identifying tumor-associated antigens and serum autoantibodies in gastrointestinal malignancies. The authors previously reported the clinical significance of antibodies against SKI and TMED5 in esophageal cancer, and antibodies against ENO1 and SSNA1 in gastric cancer. Together with these earlier observations, the results of the present study support the concept that tumor-associated humoral immunity can provide clinically relevant information for cancer detection and prognostic stratification. In this regard, s-POTEE-Ab appears to represent another promising member of the expanding panel of serological biomarkers derived from tumor-associated antigens.

Several limitations of this study should be acknowledged. First, this was a retrospective study based on samples collected at a limited number of institutions and was subject to potential selection bias and the lack of matched control populations. In particular, the use of non-age-matched healthy donors may have influenced the estimated diagnostic performance. Second, SCC-Ag and CEA were not measured in healthy donors; therefore, specificity, PPV, NPV, AUC, and logistic regression-based diagnostic performance could not be calculated for panels containing these conventional markers. Analyses involving SCC-Ag or CEA were consequently limited to positivity rates among patients. Third, the diagnostic logistic regression models combining s-POTEE-Ab and s-p53-Ab were developed and evaluated in the same datasets without internal resampling or external validation, which may have resulted in optimistic performance estimates. Fourth, the number of cases available for some subgroup analyses differed according to serum availability and completeness of clinical data, which may have affected the robustness of certain comparisons. Fifth, no formal a priori sample size calculation was performed because the cohort sizes were determined by the availability of pretreatment serum samples. In particular, the limited size of the GAC cohort may have resulted in imprecise, unstable, or potentially biased estimates in the subgroup analyses and increased the risk of type II error. Because no independent validation cohort was available, the reproducibility of the GAC findings could not be assessed. Therefore, these results should be regarded as exploratory and require confirmation in a larger, prospectively collected, independent cohort. Sixth, different cutoff strategies were used for diagnostic and prognostic analyses: the Youden index or predefined diagnostic thresholds for diagnostic assessment and X-tile analysis for survival analysis. These approaches reflected different objectives but may limit reproducibility. Seventh, the candidate-screening and public-database analyses used HNSC as an exploratory, histologically related surrogate because an ESCC-specific cohort was not available as a separately analyzable dataset in the Human Protein Atlas. Given the biological differences between HNSC and ESCC, this approach may have affected candidate prioritization, and the findings should be considered supportive and hypothesis-generating rather than direct evidence for ESCC. In addition, the ESCC cohort included both surgically and nonsurgically treated patients. Although treatment modality was included in the multivariable Cox model, residual confounding related to treatment selection, performance status, comorbidities, and patient preference cannot be excluded. Eighth, given the relatively limited number of events in some subgroups, the prognostic multivariable models may be subject to overfitting. Finally, the study was observational, and the mechanistic relationship between POTEE expression, humoral immune responses, and tumor progression remains unclear. Prospective studies with well-matched cohorts, ESCC-specific transcriptomic validation, and complete measurement of all candidate markers are required.

In conclusion, serum anti-POTEE antibodies were elevated in patients with gastrointestinal cancers and showed diagnostic performance comparable to that of s-p53-Ab. Combining s-POTEE-Ab and s-p53-Ab in logistic regression models did not clearly improve discrimination over the individual markers in the present cohorts. The evaluated serum markers showed complementary positivity among patients, but the present findings do not demonstrate synergistic effects or superiority of multimarker combinations over individual markers for diagnosis or prognostic stratification. The prognostic associations of s-POTEE-Ab were cancer-type dependent: positivity was associated with poorer overall survival in ESCC and CRAC in univariate analyses, but not in GAC, and remained independently associated with overall survival only in CRAC after multivariable adjustment. In particular, the findings in GAC remain preliminary because of the limited sample size, the potential instability of subgroup estimates, and the absence of an independent validation cohort. Large-scale prospective studies with complete marker measurement and independent validation are warranted.

## Conclusions

The present study demonstrated that serum anti-POTEE antibodies are elevated in gastrointestinal cancers and show diagnostic performance comparable to that of s-p53-Ab. The evaluated serum markers exhibited complementary positivity among patients, but synergistic effects and superiority of multimarker combinations over individual markers for diagnosis or prognostic stratification were not demonstrated. The prognostic associations of s-POTEE-Ab were cancer-type dependent: positivity was associated with poorer overall survival in ESCC and CRAC in univariate analyses, but not in GAC, and remained independently associated with overall survival only in CRAC after multivariable adjustment. The GAC findings remain preliminary because of the limited sample size and lack of independent validation. These results support further evaluation of s-POTEE-Ab as a promising exploratory serological biomarker in larger, prospectively collected, independent cohorts.

## Supplementary Information

Supplementary Material 1.

## Data Availability

The datasets generated and/or analyzed during the current study are available from the corresponding author upon reasonable request. Publicly available transcriptomic datasets used for candidate screening and survival analyses were obtained from the Human Protein Atlas and The Cancer Genome Atlas.

## Abbreviations

POTEE: POTE ankyrin domain family member E
p53: Tumor protein p53
s-POTEE-Abs: Serum anti-POTEE antibodies
s-p53-Abs: Serum anti-p53 antibodies
