# Supplementary material for: Serum anti-POTEE antibodies as a novel serological biomarker for early detection and prognostic stratification in gastrointestinal cancers
Source: BMC Cancer. 2026 Aug 10;26:1377. doi: 10.1186/s12885-026-16732-6 (PMC13749799; doi:10.1186/s12885-026-16732-6)
Supplement: Supplementary file 1 [file 12885_2026_16732_MOESM1_ESM.docx]

**Supplementary Figure 1.**

Exploratory analysis of the prognostic significance of POTEE mRNA expression in public datasets.

Kaplan–Meier curves showing overall survival according to POTEE mRNA expression levels in HNSC, STAD, and COAD datasets obtained from the Human Protein Atlas. HNSC was evaluated as an exploratory histologically related surrogate because an ESCC-specific survival cohort was not separately available. The HNSC findings should not be interpreted as direct evidence for ESCC. High POTEE expression was significantly associated with poorer prognosis in HNSC, whereas nonsignificant trends toward poorer prognosis were observed in STAD and COAD.

Supplementary Figure S2. Flow diagram of patients included in the diagnostic and marker-overlap analyses.

Serum s-POTEE-Ab and s-p53-Ab levels were available for all patients and were used for antibody-level comparisons and ROC analyses. The marker-overlap and combined-positivity analyses shown in Figures 3 and 4 were restricted to patients with complete measurements of all markers included in each panel. Complete data were available for 121 patients with ESCC, 34 of 62 patients with GAC, and 149 of 158 patients with CRAC. CEA and SCC-Ag were measured as part of routine clinical care and were unavailable in some patients because these tests had not been ordered by the treating physicians.

| Supplementary table S1. List of 226 genes highly expressed in the testis, ovary, or placenta based on the Human Protein Atlas RNA tissue expression consensus dataset. | | | | | | | |
| --- | --- | --- | --- | --- | --- | --- | --- |
| AGEC2 | SPO11 | PAGE1 | FKBP6 | LRRC74A | SEL1L2 | H2BW2 | LUZP4 |
| CCDC70 | ESX1 | ATXN3L | MAGEA10 | BANF2 | SSX1 | QRICH2 | VCY1B |
| USP26 | PIWIL4 | GH2 | C4orf17 | PLCZ1 | SYCP3 | ADAM21 | DMRTC2 |
| ALLC | SLC25A31 | CATSPER3 | TEKT5 | FAM81B | TEX29 | TRIM42 | MAGEA8 |
| TEX47 | LRRC18 | RPL10L | NOXRED1 | SSX3 | PASD1 | TBATA | SUN5 |
| GARIN2 | CBY2 | C9orf131 | ARL13A | GOLGA6L2 | TEX36 | CATSPER1 | CCDC197 |
| BEND2 | C12orf54 | SPATA31E1 | PRM3 | ZNF716 | SPDYE4 | REC114 | TFDP3 |
| FAM47A | NPAP1 | SLC36A3 | CCDC73 | H2AP | NANOS3 | DNAH17 | FAM205C |
| HBG2 | TEX43 | OR8G1 | CLEC9A | FAM24A | DPPA5 | C1orf146 | SPANXN1 |
| C16orf96 | C18orf63 | CTAGE1 | TSGA13 | MAGEB1 | DCDC2C | PNMA6E | C16orf90 |
| SPANXB1 | TEX46 | C2orf92 | TSPY8 | IQCF3 | TEX50 | PAGE2 | CCDC188 |
| TEX49 | ENSG00000259060 | GFY | SSX4 | SSX2B | CT45A1 | MEI4 | TMEM275 |
| ADAM2 | THEG | SPAM1 | SLC25A2 | TEX11 | LYZL1 | RGSL1 | IQCM |
| ZSCAN10 | CDX4 | RHOXF2 | LIN28A | FTHL17 | LARGE1 | ADAM30 | TEX52 |
| ZAN | GCNA | ANKRD30A | OOSP2 | SPATA25 | CCDC83 | LYZL2 | MEIOSIN |
| LYZL4 | HIPK4 | DPPA2 | DNAJC5G | C4orf45 | PRDM9 | SPATA48 | LITAFD |
| C16orf92 | GOT1L1 | USP50 | XAGE3 | XAGE5 | KLF17 | TEX37 | ZNF722 |
| TMEM270 | ENTHD1 | FOXR1 | DCAF4L2 | CNBD1 | TRIM60 | FMR1NB | THSD8 |
| PP2D1 | NUTM1 | PRSS55 | DDX53 | C5orf47 | ZNF479 | TEX33 | ERVW-1 |
| TMEM202 | POTEE | NANOS2 | POTEA | DUSP21 | DCAF8L2 | OTUD6A | ENSG00000284526 |
| TXNDC8 | SPANXN5 | CBY3 | FAM221B | MS4A13 | FAM205A | IZUMO3 | GMCL2 |
| KCNU1 | CDC14C | MAGEA3 | POM121L12 | SUPT20HL2 | C8orf90 | MROH5 | SMIM42 |
| TERB1 | ARMS2 |  |  |  |  |  |  |
| These genes showed significantly higher expression in the testis, ovary, or placenta than in other tissues (t-test P<0.05 and fold change >20), with no detectable expression in 95% of the remaining tissues. | | | | | | | |

| Supplementary table S2. Fifteen genes with high commonality in tumor tissues of HNSC, STAD, READ, and COAD. | | | | |
| --- | --- | --- | --- | --- |
| Gene name | HNSC(%) | STAD(%) | READ(%) | COAD(%) |
| C20orf144 | 99.2 | 99.7 | 100 | 98.2 |
| DYNLT2 | 100 | 99.7 | 100 | 99.5 |
| TMEM262 | 100 | 99.7 | 100 | 99.8 |
| ACTL8 | 86.8 | 88.2 | 97.5 | 96.8 |
| AKNAD1 | 97.6 | 95.2 | 96.9 | 93.6 |
| ERICH6B | 92.8 | 97.7 | 96.9 | 95.4 |
| C11orf94 | 96.4 | 94.1 | 88.1 | 82.7 |
| PPP1R2B | 85.4 | 99.4 | 84.3 | 77.4 |
| C1orf100 | 86.8 | 93.2 | 82.4 | 76.1 |
| CAGE1 | 99.4 | 85.6 | 82.4 | 75.9 |
| CSTL1 | 82.8 | 81.4 | 81.1 | 82.2 |
| **POTEE** | **90.8** | **99.7** | **79.2** | **69** |
| TEX19 | 80 | 77.2 | 76.7 | 77.7 |
| MMP20 | 70.5 | 74.9 | 75.5 | 70.8 |
| CCDC168 | 91.4 | 97.2 | 69.8 | 71.8 |
| The table lists 15 genes showing high commonality in tumor tissues of HNSC, STAD, READ, and COAD, defined as expression in tumor tissues in more than 60% of cases for each cancer type. HNSC, head and neck squamous cell carcinoma; STAD, stomach adenocarcinoma; READ, rectal adenocarcinoma; COAD, colon adenocarcinoma. | | | | |
